# Two‐Photon Polymerization: Fundamentals, Materials, and Chemical Modification Strategies

**DOI:** 10.1002/advs.202204072

**Published:** 2022-12-30

**Authors:** Seán O'Halloran, Abhay Pandit, Andreas Heise, Andrew Kellett

**Affiliations:** ^1^ CÚRAM the SFI Research Centre for Medical Devices School of Chemical Sciences Dublin City University Glasnevin Dublin 9 Ireland; ^2^ CÚRAM the SFI Research Centre for Medical Devices University of Galway Galway H91 W2TY Ireland; ^3^ RCSI University of Medicine and Health Sciences Department of Chemistry 123 St. Stephens Green Dublin Dublin 2 Ireland; ^4^ Advanced Materials and Bioengineering Research Centre (AMBER) RCSI University of Medicine and Health Sciences and Trinity College Dublin Dublin Dublin 2 Ireland; ^5^ CÚRAM the SFI Research Centre for Medical Devices RCSI University of Medicine and Health Sciences Dublin and National University of Ireland Galway Galway H91 W2TY Ireland; ^6^ SSPC the SFI Research Centre for Pharmaceuticals Dublin City University Glasnevin Dublin Dublin 9 Ireland

**Keywords:** 3D laser printing, direct laser writing, modification strategies, photoresists, two‐photon polymerization

## Abstract

Two‐photon polymerization (TPP) has become a premier state‐of‐the‐art method for microscale fabrication of bespoke polymeric devices and surfaces. With applications ranging from the production of optical, drug delivery, tissue engineering, and microfluidic devices, TPP has grown immensely in the past two decades. Significantly, the field has expanded from standard acrylate‐ and epoxy‐based photoresists to custom formulated monomers designed to change the hydrophilicity, surface chemistry, mechanical properties, and more of the resulting structures. This review explains the essentials of TPP, from its initial conception through to standard operating principles and advanced chemical modification strategies for TPP materials. At the outset, the fundamental chemistries of radical and cationic polymerization are described, along with strategies used to tailor mechanical and functional properties. This review then describes TPP systems and introduces an array of commonly used photoresists including hard polyacrylic resins, soft hydrogel acrylic esters, epoxides, and organic/inorganic hybrid materials. Specific examples of each class—including chemically modified photoresists—are described to inform the understanding of their applications to the fields of tissue‐engineering scaffolds, micromedical, optical, and drug delivery devices.

## Graphical abstract

This review describes the fundamentals of two‐photon polymerization (TPP) along with chemical modification strategies of TPP materials applied to medical devices, optics, cell biology, and drug delivery systems.

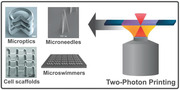

## 1 Introduction

Polymerization techniques are a staple of modern chemical production and they are applied extensively within industry for generating the items of everyday life.^[^ ^1^ ^]^ While there are numerous polymerization methods—ranging from classical processes to more complex solid‐phase methods—this review will focus on photochemical radical and cationic polymerization. Industry has benefited from the rapid production of polymers through both techniques, resulting in their extensive use in packaging, clothing, adhesives, medicinal products, over‐the‐counter medical devices, and countless other consumers products.^[^ ^1^, ^2^ ^]^ Radical polymerization is ubiquitous for the industrial production of polymeric materials and although it benefits from a rapid production rate together with a vast choice of available monomers, the reactive nature of radicals instills a lack of control in the overall process. In free radical polymerization the chain length and molecular weight of resultant polymers is impossible to predict due to the high probability of chain termination. Radical polymerization has therefore excelled where products do not require an exact molecular weight, however, precise control of polymeric chains and resultant structures has long been sought after.

Since the advent of controlled radical polymerization, polymers of consistent chain length that allow preordained molecular weights can be produced.^[^ ^3^, ^4^ ^]^ The development of radical addition fragment chain‐transfer (RAFT), atom radical transfer polymerization (ATRP), and nitroxide mediated polymerization (NMP) has pioneered the precise design of polymer structures.^[^ ^5^, ^6^, ^7^, ^8^, ^9^ ^]^ With the ability to synthesize chemically consistent and structurally defined (co)polymers, researchers were able to access well‐defined micro‐ and nanostructures, for example, micelles and vesicles mostly through self‐assembly processes. In a parallel development for the fabrication of polymer nanostructures, two‐photon polymerization (TPP) 3D photolithography has emerged to enable rapid prototyping of micro‐ and nano‐featured polymeric materials. TPP is a micro‐ to nanoscale photolithography technique that operates on the two‐photon absorption (TPA) principle to produce subdiffraction limit feature sizes. The technique uses femtosecond lasers operating in the near‐infrared (NIR) range and is applied to produce tissue‐engineered scaffolds, micromedical devices such as microswimmers and needle arrays, optical devices, and more.^[^ ^10^, ^11^ ^]^ These devices are continually evolving with higher resolution and smaller features sizes down to hundreds of nanometers becoming routinely accessible. We are now starting to see the future of TPP where the modification of structures down to the molecular level with localized functionalization is enabled by synthetic chemistry.

In this review, we present a background to the chemical processes involved in TPP and provide discrete examples of the potential uses of an array of relevant materials. The application of synthetic chemistry used to modify the chemical and physical properties of functional polymers is described along with an overview of preparative methods that have so far been successfully employed within the TPP field.

## 2 Photopolymerization

Photopolymerization is achieved through the generation of reactive species by UV, visible, or infrared light in the presence of reactive monomeric or oligomeric materials.^[^ ^12^ ^]^ Materials are generally split into two main categories for photopolymerization: radical or cationic. Radical polymerizations are achieved through the generation and transfer of reactive species through a growing chain, used commonly in the polymerization of acrylic, styrene, and other alkene‐based monomers. Cationic polymerizations are typically achieved through ring‐opening polymerization of molecules with epoxide, lactone, and cyclic ether functionalities. A material which is capable of undergoing photopolymerization is typically called a photoresist and comprise of monomeric or oligomeric material combined with a photoinitiator. However, other components are often added such as crosslinking agents, photosensitizers, and coinitiators to modify the physical and chemical characteristics of the resultant materials. Additions are often made to increase crosslinking which: i.) alters rigidity or stability to temperature and mechanical stress; ii.) reduces postpolymerization shrinkage; and iii.) eases handling by altering the viscosity of the resultant material.^[^ ^13^ ^]^

**Figure 1:**
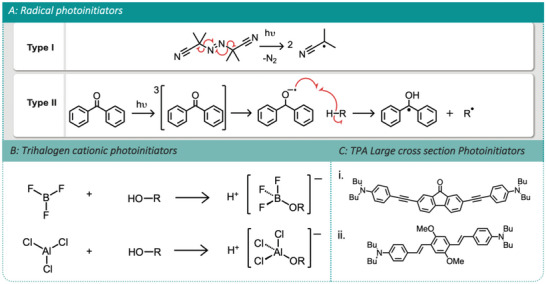
A) (I) Type I, self‐cleaving photoinitiator azo‐bisisobutyronitrile. (II) Type II, acid catalyzed photoinitiator benzophenone. B) Formation of the initiating ion pair Lewis acid from the reaction of trihalogen containing compounds with hydroxyl‐containing compounds. C) High TPA cross‐section photoinitiators: i) B3FL and ii) R1.

Photoinitiators are molecules that upon exposure to light (of the relevant wavelength) degrade to active species that are central to the make‐up of a photoresist (**Figure** **1**). While photopolymerization reactions can occur upon exposure to light in the absence of photoinitiators, their addition significantly increases the rate of reaction.^[^ ^14^ ^]^ Several classes of photoinitiators are available for both radical and cationic polymerizations (discussed below) but some common factors should be considered when choosing the appropriate one. A photoinitiator of sufficiently high cross section—a measure of its likelihood to undergo transition to an excited state on absorption of sufficient energy—increases the sensitivity of the resultant photoresist to polymerization. The UV and near infrared (NIR) cross sections are not analogous and, although most photoresist can be polymerized by either UV or NIR exposure given the absorbance of the relevant wavelengths, their efficiencies differ. This is often combined with the quantum efficiency of the initiator, which is a measure of the probability that the molecule will break down into active fragments.^[^ ^15^, ^16^, ^17^ ^]^

Photosensitizers are less commonly added to photoresists but are occasionally applied to aid the activation of photoinitiators with lower cross sections. Photosensitizers are typically molecules with large cross sections and hence easily transition to excited states which typically do not breakdown into active fragments, acting instead as carriers of triplet excited states. These excited state photosensitizers either undergo transfer of excited states or reactions with photoinitiators to generate the active species required to initiate polymerization.^[^ ^18^ ^]^

Photosensitizers and photoinitiators are regularly combined, by substituting initiators with conjugated ancillary groups to increase their TPA cross section and hence their sensitivity to TPP, rendering them high efficiency photoinitiators (Figure 1C).^[^ ^19^, ^20^ ^]^ This approach can be used to reduce the number of component parts of a photoresist enabling easier formulation.

Cross‐linkers are a staple of modern polymerizations, used to link growing chains of polymers. There are myriad methods to achieve crosslinking, from hydrogen bonding and physical entanglement to covalent linkages, depending on the structure of the polymer in question.^[^ ^21^ ^]^ Covalent linkages are the most common method and achieved through the addition of monomers characterized by the presence of multiple polymerizable handles on a single molecule such as pentaerythritol triacrylate and polyethylene glycol diacrylates. The addition of cross‐linkers allows for modification of the physical and mechanical properties of the resultant polymers. This can range from an increase in rigidity or resistance to compression, to altering the thermal stability of the resultant materials.^[^ ^22^ ^]^

Photoresists are split into two main categories: negative tone and positive tone. Both rely on a significant phase change of the exposed resist, resulting in an altered solubility compared to the unexposed resist. When exposed to an appropriate wavelength of light, negative tone photoresists are polymerized/crosslinked. The subsequent developing step only removes unpolymerized material from the photoresist unexposed to light. Conversely, areas exposed in positive tone photoresists are degraded and, on development, the unexposed area of the material remains.^[^ ^23^ ^]^ Positive tone photoresists are applied in UV photomask polymerizations, where a masking template is placed over the positive tone resist in the pattern of the required part. On exposure the unmasked material undergoes a phase change and can then be washed away leaving the template design. Negative tone photoresists are the most typical for creating TPP microstructures, however, positive tone photoresists have been applied to develop nanotrenches and microfluidic devices.^[^ ^24^ ^]^

### 2.1 The Fundamentals of Radical Polymerization

**Figure 2:**
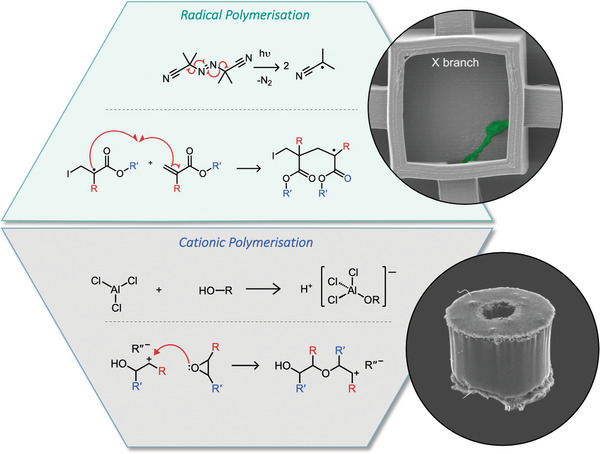
Radical polymerization: The initiation and initial propagation of radical polymerizations using AIBN and acrylic monomers, with SEM imaging of TPP produced neuronal cell scaffold using IP‐DIP photoresist from Nanoscribe (cf. Section 4.1.3). Reprinted (adapted) with permission.^[^ ^62^ ^]^ Copyright 2022, American Chemical Society. Cationic polymerization: The initiation and initial propagation steps of cationic polymerizations utilizing aluminum trichloride Lewis acid and epoxide monomers, with SEM imaging of TPP produced using epoxide doped resin (cf. Section 4.4.1). Adapted with permission.^[^ ^108^ ^]^ Copyright 2022, Springer Nature.

Radical polymerization is based on the production of radical species through thermal or light activation followed by propagation with monomers capable of carrying a radical (**Figure** **2**).^[^ ^25^ ^]^ Radical initiators are compounds that degrade under mild conditions to release radical species, which generate monomer‐initiator radicals that propagate the polymerization process.^[^ ^26^ ^]^ Radical initiators can be divided into thermally activated and photoactivated initiators, however, photoinitiators will be the primary focus here. There are multiple photoinitiators which find common use in the field of polymer chemistry. Broadly, they include two main classes: self‐cleaving initiators, which form radicals without the need of a coinitiator (Norrish Type I) and hydrogen‐transfer type compounds (Norrish Type II), which are reliant on the abstraction of a proton from a coinitiator species to form a radical and single electron transfer from dye molecules acting as intermediates between photosensitizers and photoinitiators (Figure 2).^[^ ^27^, ^28^ ^]^ Azo‐initiators are a prominent class of self‐cleaving initiator that rely on either thermal or photoinduced release of nitrogen and result in two carbon radicals. The most common initiator from this family, azo‐isobutyronitrile (AIBN), is used extensively in polymer chemistry. AIBN proceeds through homolytic cleavage and release of nitrogen, forming two cyano‐functionalized alkyl radicals. On the other hand, hydrogen‐transfer type initiators such as benzophenone rely on the abstraction of a labile proton from a coinitiator species, such as protonated tertiary amines.^[^ ^29^ ^]^ A wide range of photoinitiators are commercially available, tailored for different wavelengths of activation, stability, solubility, and suitability to the polymerization process.

Free radical polymerization is the simplest method and once initiated proceeds through propagation with the radical typically located on the growing chain. This is followed by termination through a combination of free radicals or disproportionation of free radicals resulting (in the case of vinyl monomer systems) in alkane and alkene terminated polymeric chains. The method results in rapidly propagating, highly polydisperse polymer chains and is the least controlled method. Better control over molecular weight, polydispersity, and end groups can be achieved through thermally initiated controlled radical polymerization. Controlled radical polymerization differs from free radical polymerization through reduced chain termination. Chain initiation is faster than chain propagation and, as a result, a constant number of growing chains is maintained during the polymerization process.^[^ ^3^, ^5^ ^]^ Recently, photoinitiated controlled radical polymerizations have been elucidated and although these have not been applied in TPP they offer significant potential.^[^ ^30^ ^]^

### 2.2 The Fundamentals of Cationic Polymerization

Cationic polymerization relies on transferring a cationic charge from growing polymeric chains to nucleophilic monomers or oligomers. The first reported examples of cationic polymerization were in the 1700s, far predating radical polymerizations.^[^ ^31^ ^]^ Cationic polymerization has been applied industrially to produce butyl rubbers, hydrocarbon resins, and epoxy resins.^[^ ^32^ ^]^ It functions through the generation of a cationic initiating species which undergo nucleophilic attack from a monomer, transferring the cationic charge to the monomer species and causing propagation. The method relies on the monomers to act as a nucleophile and is limited to the use of vinyl monomers with electron‐donating substituents and heterocycles; cationic polymerization is thus separated into two main categories: vinyl and ring‐opening polymerization.^[^ ^33^ ^]^

Cationic initiators are typically Lewis acids, such as boron trifluoride and aluminum(III) chloride, relying on the addition of water or alcohols to form the initiator ion pair (Figure 2). The initiator can then induce a positive charge on the monomer species through nucleophilic attack of the monomer against the Lewis acid proton resulting in a protonated monomer–Lewis acid anion pair. The polymerization of vinyl‐containing monomers functions by generating carbocations, followed by nucleophilic attack by electron‐donating substituted vinyl monomers.^[^ ^33^ ^]^ Ring‐opening polymerization functions in much the same manner, except heteroatoms such as oxygen or nitrogen, hold the cationic charge in equilibrium. Polymerization then propagates through the carbocation, assisted by the ring strain of the monomer shifting the equilibrium to the carbocation.

As with radical polymerization, initiation of cationic systems can be achieved through either thermal or photochemical means. Photochemical initiators—typically called photo‐acids—produce Lewis acids upon excitation with light. Multiple examples of UV and visible light initiator systems exist with some of the earliest photoinitiators based on sulfonium compounds or aryl iodonium salts.^[^ ^34^ ^]^ Cationic polymerization can also undergo controlled processes such as living polymerization. With the lack of water as a coinitiator, initial living cationic systems relied on so‐called cationogens. The abstraction of halogens such as chlorine from *tert*‐butyl chloride used diethyl aluminum chloride to form the initial Lewis acid.^[^ ^31^ ^]^ Similar systems were achieved using iodide/iodine to initiate the polymerization of isobutyl vinyl ether and complexes of boron trichloride with tertiary organic esters for the polymerization of isobutylene.^[^ ^35^, ^36^ ^]^ Cationic systems can be tailored for increased control by choosing the Lewis acid (and hence counter ion) for the growing cationic chain and careful selection of solvents. Living cationic polymerization was also achieved with metal halide complexes in conjunction with a Lewis base, and systems applying iron(III) chloride and tin(IV) chloride with weak bases identified to promote ultrafast polymerization.^[^ ^37^ ^]^

Another form of controlled cationic polymerization is cationic RAFT (C‐RAFT) and early examples applied thiocarbonylthio reagents with the addition of ppm concentrations of triflic acid for the polymerization of isobutyl vinyl ether.^[^ ^38^ ^]^ Since the advent of the original RAFT method, systems controlled with visible light have been developed using 2,4,6‐tri‐(*p*‐methyoxyphenyl)pyrylium tetrafluoroborate with RAFT agents or metal complex based systems.^[^ ^39^ ^]^ Such controlled polymerization seeks to overcome the high reactivity of the carbenium ions due to the system's sensitivity to impurities that cause rapid propagation and highly polydispersed polymers. Terminations of cationic systems typically occur from the interaction of the growing cationic chain with an anionic fragment of the original counter ion, often resulting in the reduction of the Lewis acid counterion. In cases involving metal‐halogen Lewis acids, the resulting polymeric chain is capped with a halogen, and the metal complex is reduced.

## 3 Two‐Photon Polymerization

**Figure 3:**
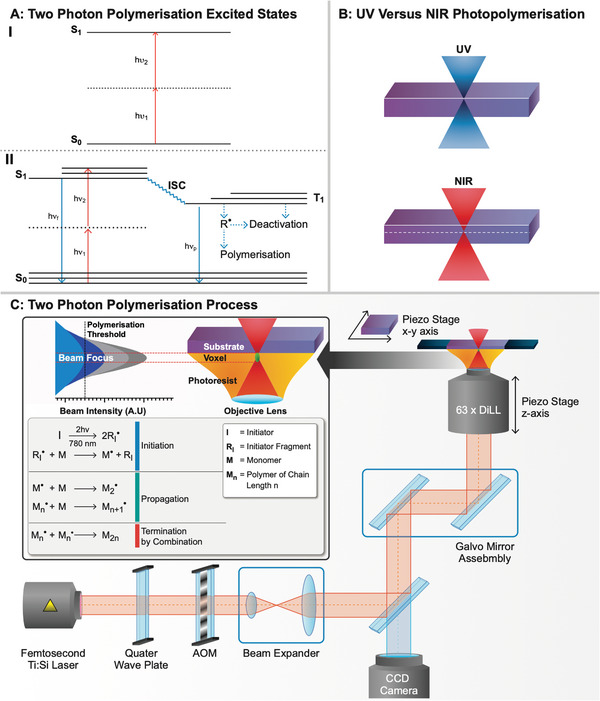
A) (I) Two‐photon absorption leading to the excitation of an electron to an excited state through a simultaneous absorption and a virtual, short‐lived intermediate state. (II) Initiation of the TPP process, displaying the competing processes to radical generation (R•), through emissive (solid blue line) fluorescence (*hν_f_*) and phosphorescence (*hν_p_*) and nonemissive (dashed blue line) routes. (Adapted from Sun and Kawata) B) (I) UV photopolymerization displaying low penetration and high surface interaction with a photopolymerizable material. (II) NIR photopolymerization displaying minimal surface interaction and increased penetration when compared with UV. C) TPP system setup for printing in dip‐in laser lithography (DiLL) mode.

Two‐photon polymerization has come to the fore in materials and polymer chemistry and several comprehensive reviews describing the photophysics of the method are available.^[^ ^40^, ^41^, ^42^, ^43^ ^]^ TPA was first theorized by Maria Goeppert Mayer in her doctoral thesis in 1931 and experimentally observed in 1961 by Kaiser and Garrett during the excitation of CaF_2_:Eu(II) crystals through two‐photon adsorption of red light followed by blue fluorescence.^[^ ^44^ ^]^ TPA functions by the principle that materials can transition to excited states through the absorption of two photons in a simultaneous manner, allowing the energy of a single photon to be less than the energy gap between excited states (**Figure** **3**A). This relies on the initial absorption of the first photon to promote an electron to an intermediate excited state with lifetimes in the femtoseconds.

Since its discovery, TPA has found application in the emergence of TPA spectroscopy, optical data storage, tissue and cellular imaging. The application of TPP lithography—often referred to as direct laser writing (DLW)—for microscale nanoresolution 3D printing was first applied in 1965 by Pao to the polymerization of styrene in a two‐photon process.^[^ ^45^ ^]^ Due to the lifetime of virtual excited states the light sources typically used for two‐photon polymerizations are typically femtosecond lasers capable of dosing the photopolymerizable resin with sufficient energy within this lifetime.

The light sources typically used are NIR femtosecond lasers operating at a longer wavelength than UV light. NIR sources have the added benefit that since many materials in current use are transparent to NIR light, resulting in minimal interaction of the material with the incident light other than at the focal volume (Figure 3B).^[^ ^46^ ^]^ The use of NIR sources is however dependent on the use of initiators or sensitizers absorbing in the NIR range (700–800 nm). NIR light is also within the so‐called “biological window” with most biological tissues being transparent in the NIR range, allowing for the potential application of in vivo printing of structures for cell architectures.^[^ ^47^, ^48^ ^]^ These factors allow the printing of arbitrary shapes in the 3D environment, giving access to geometries previously impossible using traditional lithography methods. The technique was not established until the advent of lasers, which can supply sufficient photon flux density (and hence light intensity) to initiate efficient polymerizations.

The photon flux density (units mmol m^−2^ s^−1^) is a function of the power output of the laser, the focal volume the laser spot is focused through, and the duration the laser is applied to a specific volume. As a result, achieving efficient polymerization by TPP processes requires the optimization of several system parameters. This includes the write speed of the system which is inversely proportional to the laser dose achieved and the laser power which is proportional to the same. These two factors coupled with the focusing of the laser source are responsible for the laser dose supplied to any given volume, measured in photon flux density. Altering materials will change the required dose to achieve efficient and highly resolved microstructures by two‐photon polymerization.

Fine features are generally best suited to lower powers due to the nonlinear nature of TPP absorption and due to the possibility for overexposure and damage to the resulting features when focusing the laser source into a smaller volume. This damage can occur when any given area is exposed to the laser source for a relatively long duration, causing a build‐up of heat and evolution of gases typically seen as bubbling during a print and indicative of poorly optimized dosing conditions.

When the laser source is focused into the photoresist, polymerization occurs through the excitation of initiating species to a singlet excited state followed by intersystem crossing (ISC) to a triplet excited state and decaying through the cleavage of bonds to form radical species in the case of radical polymerizations (Figure 3AIII). However, this process competes with deactivation through fluorescence, phosphorescence, and initiator and radical quenching. The quenching of the initiator in a triplet state and subsequent radicals is used as a method to prevent the uncontrollable propagation of polymerization throughout the photosensitive material and can typically be achieved with dissolved oxygen present in the photopolymerizable material or through the addition of radical quenching compounds.^[^ ^49^ ^]^ This control over the propagation aids in the polymerization of small volumes allowing access to fine feature resolution when optimized. The smallest possible volume which can be polymerized by TPP is known as a voxel. The size of the voxel produced by TPP is beyond the capabilities of a single photon process, with subdiffraction limit feature sizes possible (see cf. ref. [13] for further details on the diffraction limit, subdiffraction limit resolution, and nonlinear nature of TPP^[^ ^13^ ^]^). The size of a voxel is optimized when dosing the photoresist only fractionally above the polymerization threshold as shown in Figure 3, as well as using high numerical aperture optics, focusing the center of the beam into a smaller area.^[^ ^13^ ^]^ Voxels are naturally extended in the *z*‐axis due to the contraction and expansion of the beam before and after the focus point, p to the *z*‐axis, leading to a mirrored volume above and below the point of focus which will be polymerized, while the *x*–*y* geometry of the voxel is not mirrored. As a result, higher resolutions are typically achieved in the *x*–*y* axis over the *z*‐axis. In general, single‐photon polymerizations are limited to feature sizes of half the wavelength of the incident light while TPP can feature sizes below 100 nm using a 780 nm source.^[^ ^50^ ^]^ Improvements of the radical quenching ability of TPP resins have since been used to improve resolution capabilities and has enabled resolution below 100 nm.

TPP systems are typically set up as “inverted microscopes” using microscope objective lenses to focus the femtosecond laser source into the desired region of the photoresist to induce photopolymerization. The choice of lens dictates the resolution capabilities and processing times of polymerizations, with higher magnification objectives leading to much higher resolution microstructures at the cost of increased processing time. Instruments can be set up for printing in several different modes, from dip‐in laser lithography (DiLL) to oil immersion lithography or air interface lithography. The DiLL mode uses the immersion of the objective into the photoresist being printed in, finding the photoresist–substrate (ITO‐coated slide, silicon wafer, etc.) interface as the origin of the print. The photoresist is applied to the substrate, inserted into a holder, and placed over the objective lens inverted (Figure 3C) with the system printing in the negative *z*‐axis direction. Oil immersion uses an inert oil in contact with the objective lens favoring the photoresist to prevent potential damage to the objective. The oil used must be matched to the substrate's refractive index through which microstructures are being printed to avoid unnecessary diffraction. This method is typically applied with photoresists containing solvent, strong acid or base to protect the objective from degradation. The immersion oil is placed on the objective side of the substrate while the photoresist is placed on the opposite causing the system to print through the substrate. This restricts oil immersion lithography to thinner substrates and results in a reduced *z*‐axis range. The print origin is taken at the substrate–photoresist interface, with the system printing in the positive z‐axis direction. Air interface lithography uses the air–photoresist interface; however, reflection interference from the photoresist surface can cause problems. As such, angling the objective with the substrate has enabled the freeform printing of optical devices.^[^ ^51^ ^]^

## 4 Unmodified Photoresists and Their Applications

Commercially available photoresists are used extensively in TPP and are the primary substrate used in the rapid prototyping of novel microstructures. With multiple materials commercially available, a wide variety of devices can be produced without labor‐intensive modification of existing or the production of custom formulated resins. These include, for example, optical devices, medical devices, microfluidic devices, and cell scaffolds. Therefore, the ready availability of photoresists has enabled TPP to become a state‐of‐the‐art field where preparative chemistry expertise does not generally provide a barrier to the development of novel microstructures.

### 4.1 Acrylates

**Figure 4:**
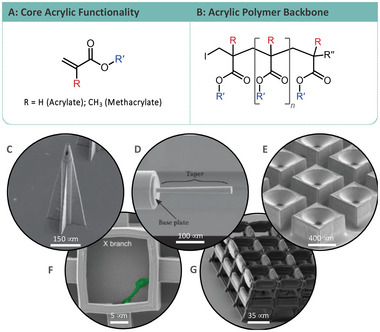
A) Core acrylic functionality. B) Acrylic polymer backbone. C) Acrylic microneedle. Adapted with permission.^[^ ^53^ ^]^ Copyright 2022, Elsevier. D) Microfiber optic tapers for beam expansion. Adapted with permission.^[^ ^55^ ^]^ Copyright 2022, Elsevier. E) Micro aerosolizer. Adapted with permission.^[^ ^59^ ^]^ Copyright 2022, Institute of Electrical and Electronics Engineers. F) Cell scaffolding for neuronal networks. Adapted with permission.^[^ ^62^ ^]^ Copyright 2022, American Chemical Society. G) Acrylic cell scaffolding. Adapted with permission.^[^ ^61^ ^]^ Copyright 2022, Wiley.

Acrylate‐based photoresists are popular throughout TPP and are used extensively to produce medical devices, cell scaffolds, and negative tone prototypes for casting due to their versatility, rapid processing, and ease of development.^[^ ^52^, ^53^ ^]^ Poly(methyl methacrylate) (PMMA) resins have found use in optical applications such as microfiber optics and have since found application in TPP to produce optical components. Acrylate‐based photoresists contain the same acrylate functionality with substituents on the R and R′ positions (**Figure** **4**A,B), with the R′ position more extensively used to tailor the properties of the polymerized material. The production of acrylate derivatives is regularly achieved through esterification or amidation of acrylic acid, acryloyl chloride, and acrylic anhydride with suitable alcohols or amines. Acrylic‐based polymers are produced through radical polymerization (Figure 2), however, non‐TPP methods have been used for ionic polymerization of acrylate block copolymers.^[^ ^54^ ^]^ Multiple examples of commercially available acrylic‐based resins for use with TPP can be found in literature (Figure 4C–G), primarily due to the relative ease of processing acrylate photoresists, high‐resolution capabilities, and mechanical robustness.

#### 4.1.1 Acrylate‐Based Optical Devices

Commercially available acrylate resists such as the IP range of photoresists from Nanoscribe GmbH have been used extensively for rapid prototyping and the production of numerous devices. This range of photoresists are a tailored mix of acrylic monomers for TPP, optimized for their optical properties, resolution capabilities, and minimize printing errors such as shrinkage. The applications of commercial resists span from optical components through to medical devices. Freeform micro‐optics have been constructed using IP‐S acrylate‐based photoresist for optical coherence tomography (OCT) fiber optic probes (Figure 4D).^[^ ^51^, ^55^ ^]^ The fabrication of freeform micro‐optics has been impossible with previous production methods since overcome with the advent of TPP capable of producing arbitrary 3D structures with resolutions allowing for microstructures with optically smooth surfaces. In addition, side view micro‐optics produced by TPP, operating via total internal reflection, have the potential for application with biological imaging, offering minimal attenuation of the probe beam at the 1300 nm wavelength used for OCT.

**Figure 5:**
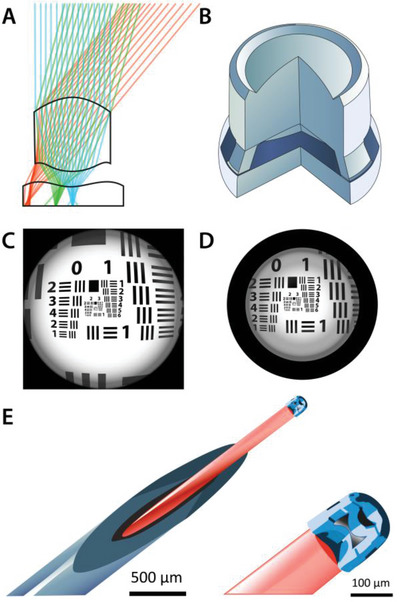
Illustration of micro‐optics constructed using IP‐S acrylate‐based resin. A) Proposed design of doublet micro‐optic. B) SEM image of doublet lens printed with 90° section to display internal lens doublet. C) Simulated image of the resolution test chart. D) Resolution test chart observed using doublet lens printed using IP‐S. E) IP‐S triplet micro‐optic arrested on the end of a microfiber optic cable suspended in a needle. Illustrations generated from ref. [56].

Other commercially available resin examples can be found, with IP‐DIP photoresist designed for DiLL being used to produce optically smooth surfaces and has been shown to fabricate complex, multi lensed optics on the micrometer scale.^[^ ^56^ ^]^ These multilensed optics have been designed (**Figure** **5**A), constructed by TPP (Figure 5B) and then tested using USAF optical resolution benchmarks (Figure 5C,D). Such micro‐optics have the potential for applications in medical imaging, reducing the invasiveness of medical procedures through placement at the termini of microfiber optical devices for keyhole surgery (Figure 5E).

#### 4.1.2 Acrylate‐Based Drug Delivery Devices

The use of acrylate‐based photoresists also has potential medical applications in drug delivery. Implantable microcage devices have been reported for use as slow release of a drug after implantation. These devices have been printed using IP‐S acrylate‐based photoresist, designed at 900 µm tall with a 340 µm outer diameter (300 µm inner diameter) with 5 µm perforations spaced 5 µm apart. These devices, loaded with agarose and fluorescent dye, displayed the ability to elute 40% of their load over 24 h. The printed microstructures have also shown the robustness to implantation in the murine brain both ex vivo and in vivo and successfully tested for the slow release of biocytin.^[^ ^57^ ^]^ Similarly, microreservoirs have been designed to elute anti‐inflammatory drugs when stimulated with electric current. The structures, constructed from IP‐L 780 photoresist were designed as microtubes in which dexamethasone was loaded in a 4:1 ratio with glycerine, using the response of titanium‐polypyrrole/poly(lactic‐*co*‐glycolic) acid to stimulate the release of the drug molecule from the glycerine matrix.^[^ ^58^ ^]^ Larger scale application for IP‐S photoresist has been demonstrated with the development of improved nozzles for drug aerosolization from inhalers (Figure 4E). These nozzles boast high robustness to repeated 35 bar differential pressures and decrease in spray generation time compared to the current state of the art Rayleigh jet device used in current inhalers.^[^ ^59^ ^]^ TPP has also been used for the production of master structures to be used in PMDS micro molding for the production of a stand‐alone device consisting of microneedles attached to a flexible reservoir which on compression will inject its drug load through the microneedle array.^[^ ^60^ ^]^

#### 4.1.3 Acrylate‐Based Cell Scaffolding Devices

Tissue engineering type structures have been produced via a TPP curable resin consisting of several acrylic functionalized materials. While acrylic‐based materials are not regularly used for cell scaffolding mediums, examples are present within literature.^[^ ^61^, ^62^, ^63^ ^]^ passivation of acrylic polymers with aluminum oxide or the inclusion of an acrylic handle on the polylactide backbone has allowed the development of biocompatible and in some cases biodegradable polymers.^[^ ^62^, ^63^ ^]^ The simple wood stack style scaffolds and more complex sea‐shell type and enclosed channel type structures have showed prominent efficacy in the proliferation of neuronal cells and supports 3D cell growth. It was noted that neuronal fiber thickness could be tailored through the customization of the distance between microstructure features, allowing more robust cell growth to be achieved (Figure 4F). The biodegradability of the resulting scaffolds coupled with their ability to allow proliferation of neuronal cells, including an ability to tailor fiber thickness, shows potential application in the delivery of cell for regenerative medicine.^[^ ^63^ ^]^ This effective proliferation of neuronal cells has further benefitted from the detection of spontaneous synaptic currents from neuronal cells grown within the enclosed channel type scaffolds.^[^ ^62^ ^]^ This furthers the potential use of these types of scaffolds from enabling more efficient methods of culturing cells to providing potential test beds for myriad drug candidates including treatments for Parkinson's and Alzheimer's diseases.^[^ ^62^, ^64^ ^]^ Similar acrylic cell scaffolds have been applied for bone‐growth applications and have the potential to become implantable scaffolds for bone healing (Figure 4G).^[^ ^61^ ^]^

Other acrylic functionalized polycaprolactone scaffolds have demonstrated potential as effective implants in porcine models of retinitis pigmentosa. Here, favorable results in a one‐month trial were observed with no evidence for inflammation, toxicity, or infection. The goal of this work is to now provide cell based treatments for retinal degenerative blindness.^[^ ^65^ ^]^

### 4.2 Hydrogels

**Figure 6:**
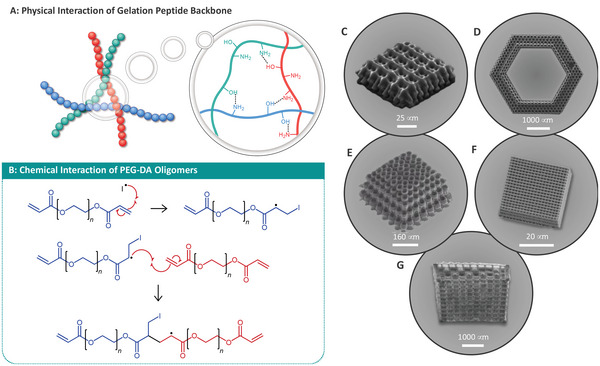
A) Physical interaction between peptide backbone of gelatin type hydrogels, hydrogen bonds between amine and carboxylate groups. B) Chemical interactions of a hydrogel consisting of PEG‐DA oligomers with *n* number of repeating glycol units. C–G) Examples of hydrogel cell scaffolding. SEM images adapted with permission. (C)–(G) Adapted with permission.^[^ ^69^ ^]^ Copyright 2022, American Chemical Society; Adapted with permission.^[^ ^64^ ^]^ Copyright 2022, IOP Publishing; Adapted with permission.^[^ ^79^ ^]^ Copyright 2022, Elsevier; Adapted with permission.^[^ ^76^ ^]^ Copyright 2022, American Chemical Society; Apadted with permission.^[^ ^78^ ^]^ Copyright 2022, Multidisciplinary Digital Publishing Institute.

Hydrogels have been used extensively to produce biocompatible devices, including tissue and cell scaffolding to develop natural 3D environments for natural cellular niches. Hydrogels are polymeric matrices capable of water absorption, with water contents similar to that of living tissues. The formulation of a hydrogel is dependent on the presence of hydrophilic groups within the polymeric network, allowing for the infiltration and retention of aqueous media within the matrix by hydrogen bonding. Hydrogel networks are formed through two main avenues: physical and chemical interactions. Physical interactions rely on the hydrogen bonding and entanglement of individual polymeric chains to form a physically bound network (**Figure** **6**A).

In contrast, chemical methods rely on the covalent or ionic fixation of monomeric, or more regularly, oligomer hydrogel chains through reactive end groups such as acrylates (Figure 6B).^[^ ^66^ ^]^ Covalently bound hydrogels are commonly used in TPP with poly(ethylene glycol)diacrylate (PEG‐DA) oligomers being employed as a starting material. Hydrogels offer potent biomimetic qualities that present a soft viscous nature with low cytotoxicity. Hydrogels, however, suffer from low mechanical stability and hence are regularly subjected to efforts to increase their crosslinking.

#### 4.2.1 Hydrogel‐Based Drug Delivery Devices

Due to the porous nature of hydrogels, their ability to swell and absorb small molecules means they are potent drug delivery devices, routinely used within polymer chemistry.^[^ ^67^ ^]^ Acrylate crosslinked hydrogels, such as PEG‐DA woodpile structures, have been applied in the design and production of drug‐eluting porous materials modeled using a Rhodamine B fluorescent dye as a drug analogue.^[^ ^68^ ^]^ These hydrogels were photopolymerized in the presence of Rhodamine B thereby encapsulating the fluorophores within the hydrogel matrix. The elution of the Rhodamine B in water was monitored over a week, and a positive correlation was found between the pore size and the release rate of the fluorophore from the hydrogel matrix. These hydrogels were also demonstrated to be noncytotoxic to a range of cell lines and may be applied in passive release drug platforms such as drug‐eluting stents.

#### 4.2.2 Hydrogel‐Based Cell Scaffolding Devices

Using a number of materials ranging from simple PEG hydrogel and acrylate functionalized gelatin materials, to custom protein based resists, simple model cell scaffolds have been designed and have demonstrated their noncytotoxic nature with countless cell lines (cf. Figure 6C–G for examples of cell scaffolds of varying topology).^[^ ^69^, ^70^, ^71^, ^72^, ^73^, ^74^, ^75^, ^76^ ^]^ A large body of work has been conducted using simple structures such as woodpiles and cylinders as the scaffold basis, with simplistic measures for culturing cell lines on the resultant microstructures. However, for applications in the field of tissue engineering, precise transfer, and placement of cells within a scaffold offers the next steps in the production of viable tissue engineering methods. PEG‐DA hydrogels have been used and applied in the laser‐induced forward transfer (LIFT) of endothelial cells and smooth muscle cells into a TPP produced porous scaffold.^[^ ^77^ ^]^ This type of hydrogel cell scaffolding is intended to replace previously generated porous materials by producing accurately known and reproducible scaffolds. Coupled with LIFT, TPP produced cell scaffolding allows for more accurate mimicking of multicellular tissue constructs such as arterial walls, accurately locating cells into the hydrogel scaffold.^[^ ^77^ ^]^ Biocompatible cell scaffolds have also been designed for their biodegradability, intending for the initial structuring and constrained proliferation of given cell lines for the creation of artificial tissue structures.^[^ ^78^ ^]^ The biocompatibility of cell scaffolds has been improved through the tailoring of photoinitiators used and the postprocess extracting of unreacted photoinitiators.^[^ ^79^ ^]^ For example, the development of water‐soluble photoinitiators has allowed for the improvement of the noncytotoxic nature and has allowed for the production of cell scaffolds around *Caenorhabditis elegans* (*C. elegans*) thereby capturing them in a woodpile structure.^[^ ^48^ ^]^ This method has also been demonstrated for the in situ TPP production of a hydrogel that encapsulated L929 mouse fibroblast cells.^[^ ^80^ ^]^ Similarly, TPP has been used for to print hydrogel architectures inside giant unilamellar vesicles without damage them.^[^ ^81^ ^]^ These methods pave the way for providing cell entrapment and tissue engineering platforms suitable for biological and medical analysis.

**Figure 7:**
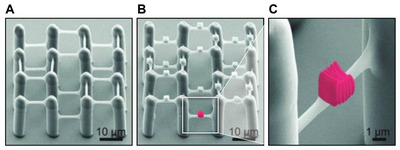
A) Cell scaffold constructed using PEG‐DA based hydrogel. B,C) Scaffold decorated with Ormocomp blocks for cell‐specific binding. Reproduced with permission.^[^ ^52^ ^]^ Copyright 2022, Wiley.

While their noncytotoxic properties have been repeatedly demonstrated, the low mechanical stability of hydrogels has led to the need for further crosslinking to allow for more stable devices to be produced. Pentaerythritol *tetra*‐acrylate (PETA), a popular cross‐linker for acrylic resins, has been incorporated into PEG‐DA hydrogels, resulting in vastly improved mechanical stabilities (**Figure** **7**A). As PEG‐DA hydrogels inhibit cell and protein adhesion, the same group has achieved 3D patterned cell scaffolds using two‐step lithography, creating a spatial resolution scaffold from PEG‐DA/PETA, followed by incorporation of Ormocomp (Figure 7B,C), a type of organic/inorganic hybrid (OIH) polymer which is discussed below (Section 4.3). Here, Ormocomp acts as a cell‐binding platform and, using selective incorporation within the main scaffold, achieves a spatial and temporal resolution of a particular cell culture.^[^ ^52^ ^]^ For further detail of biocompatible TPP cell scaffolding, Raimondi et al. provide a comprehensive review on the subject.^[^ ^82^ ^]^

#### 4.2.3 Hydrogel‐Based Microswimmers

Other hydrogel materials constructed from noncytotoxic materials such as gelatin methacryloyl (Gel‐MA) have been used to produce microrobots. For example, microswimmers decorated with magnetic nanoparticles have been produced via TPP copolymerization of Gel‐MA with PEG‐DA to create mechanically robust, biodegradable microrobots for selective drug delivery, surgical, or diagnostic applications.^[^ ^10^ ^]^ Once administered to a patient, these helically shaped microswimmers can be propelled through media, demonstrated in bovine serum albumin (BSA) glucose PBS solution, using a rotating magnetic field.^[^ ^10^ ^]^ These microswimmers showed advantageous properties over similar structures constructed from rigid materials by TPP, discussed below, due to their soft nature. This allowed a slightly increased swimming velocity in comparison to rigid material counterparts. The biodegradation of these microswimmers was achieved with collagenase (type II), cleaving the peptide bonds in the gelatin backbone, and further demonstrated with cell‐mediated degradation over a week.^[^ ^10^ ^]^ Microswimmers with internal cargo capacity have also been developed for their potential as theranostic devices. Constructed using Gel‐MA with iron oxide superparamagnetic nanoparticles, they boast the ability to deliver therapeutics as well as antibody labeling microparticles upon enzymatic mediated degradation or in another example upon irradiation with UV light for their application in the treatment and imaging of cancers.^[^ ^83^, ^84^ ^]^ Microrotors are another structure designed as potential self‐propulsion and have been reviewed by Otuka et al. (cf. ref. [33]). Using ferromagnetic components attached to one of three rotors blades, magnetic fields are used to impart rotation and in turn propulsion to the device.^[^ ^41^ ^]^

### 4.3 Organic/Inorganic Hybrids

**Figure 8:**
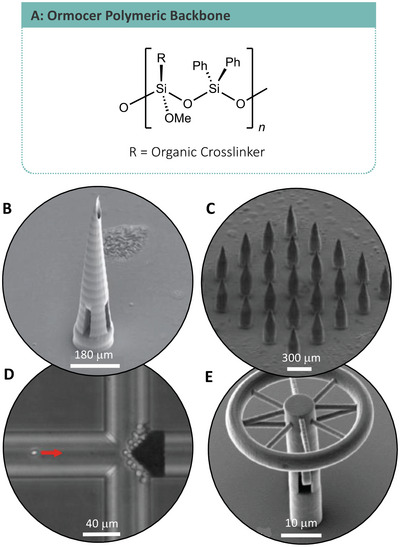
SEM images: A) Organic/inorganic hybrid backbone of ORMOCER polymers. B) Ormocer microneedle. Adapted with permission.^[^ ^87^ ^]^ Copyright 2022, American Scientific Publishers. C) Microneedle array. Adapted with permission.^[^ ^72^ ^]^ Copyright 2022, IOP Publishing. D) Microfilter for blood plasma separations. Adapted with permission.^[^ ^103^ ^]^ Copyright 2022, Royal Society of Chemistry. E) Optical microresonator for studying light matter interactions. Adapted with permission.^[^ ^104^ ^]^ Copyright 2022, Optica Publishing Group.

OIH polymer networks first found relevance in the industrial environment in the 1940s. They are typically constructed from inorganic metal alkoxides forming metal oxide polymeric matrices, through metal–oxygen–metal or metal–hydroxyl–metal bonds, typically formed through elimination reactions. Commonly, silicon oxides are used as the inorganic framework for such hybrids (**Figure** **8**A); however, the materials can be prepared from carbonates, phosphates, and chalcogenides. The organic part of the hybrid is typically formed through the attachment of organic molecules through organo‐silicon alkoxides or halides (network modifiers) or as organic polymer chains (network formers).^[^ ^85^ ^]^ In TPP, radical initiators can be used for the in situ generation of organic network formers through addition reactions. OIHs have become prevalent in medical devices such as microneedle arrays and cell culture devices due to their biocompatibility, ultralow shrinkage, and allowance for cell and biomolecule adhesion.^[^ ^86^ ^]^ However, the requirement for post‐baking steps has negatively affected the widespread use of OIHs in the production of microstructures, with many opting to use hydrogels or acrylates which boast fewer postprocessing requirements.

#### 4.3.1 Organic/Inorganic Hybrid‐Based Microneedles

Extensive research has been conducted in the area of microneedle development due to the distinct advantages microneedles offer to unskilled application and administration of drug delivering or blood sampling patches.^[^ ^72^, ^87^, ^88^, ^89^, ^90^, ^91^ ^]^ OIHs have been used to produce microneedle arrays as the next step toward such transdermal drug delivery systems. Microneedles are needles formed on the micron scale, which can be administered to patient skin and effectively penetrate the keratinized dermal layers to allow drug administration to the dermis (Figure 8B,C). Ormocer US‐S4, a methacrylate modified OIH also known as Ormocomp, has found applications within TPP for cell interface technologies. TPP has allowed for the production of microneedles at sizes previously unobtainable by traditional manufacturing methods and Ormocomp microneedles have been tested for their biocompatibility with human epidermal keratinocyte cells and their ability to penetrate dermis based on porcine skin. The use of a methacrylated OIH resulted in modified stress–strain behavior, measured against a polytetrafluoroethylene surface, indicating the potential for modifying the mechanical behaviors of OIH type materials with organic additives.^[^ ^87^, ^92^ ^]^ Similarly, a group has combined the use of Ormocomp fabricated microneedles with a PMMA microfluidic device for the injection of therapeutics, and potential application for the sampling of biological fluids.^[^ ^91^ ^]^

Ormocer microneedles have also been produced via TPP micromolding technique in which a PEG‐DA master was fabricated through TPP, followed by creating a negative silicon elastomer mold. Ormocer microneedles were then cast from the resulting elastomer mold, cured using an ultraviolet–visible (UV–vis) source. While Ormocer has been demonstrated as a noncytotoxic material demonstrated against human epidermal keratinocytes, the resulting materials were coated with antimicrobial silver eliciting antimicrobial effects against *Staphylococcus aureus* (*S. aureus*).^[^ ^89^ ^]^

#### 4.3.2 Organic/Inorganic Hybrid‐Based Cell Scaffolds

Ormocomp cell scaffolds have been developed with the ability to support a numerous cell lines, demonstrating ample biocompatibility for potential cell culture and tissue engineering applications, including stem cell proliferation and differentiation.^[^ ^52^, ^92^, ^93^, ^94^, ^95^, ^96^ ^]^ An example with B35 neuroblast‐like cells and HT1080 epithelial‐like cells with ORMOCER scaffolds reportedly allow for 100% cell viability over 48 h, with no notable difference at 96 h compared to polystyrene and extracellular matrix substrates. The scaffolds were designed as interlocking units, described as Lego‐like, and offer the potential for application in tissue engineering.^[^ ^92^ ^]^ Such cell scaffolds offer a potential increase in the cell density capable of being cultured in a single flask, with a notable 67% increase in cell density reported by Raimondi et al.^[^ ^97^ ^]^ Ormocomp (introduced in the previous section) has also been applied to a PEG‐DA cell scaffold. PEG‐DA, without additives, prevents cell and protein adhesion and hence can be applied to spatially arrange cells without unwanted adhesion. Ormocomp was further applied in a separate lithography step to include cell attachment “nodes” for 3D spatial organization and binding of cells, demonstrating control over cell shape.^[^ ^52^ ^]^

Cell morphology has also been studied on Ormocomp scaffolds, specifically focusing on the effect of actin microfilament bundles. Here, Ormocomp fiber scaffolds with individual fiber diameters of 6–9 µm were printed, and fibroblast (NIH‐3T3) and epithelial (MDCK) cell lines were then cultured. The straight patterned actin bundles of the NIH‐3T3 cells were noted to wrap around single fibers, elongate along individual fibers, and attach to multiple fibers of the scaffold. The circular filament actin of the MDCK cells resulted in their pronounced elongation along the length of individual strands and the wrapping of cells around strands.^[^ ^98^ ^]^ The same group adapted this scaffold to study contractile forces by fibroblast cells based on the deviation of single Ormocomp fibers.^[^ ^99^ ^]^ This device and approach may predict cell morphologies and tailor cell scaffold systems to further develop tissue‐engineered platforms.

#### 4.3.3 Organic/Inorganic Hybrid‐Based Microfluidic Channels

The development of microfluidic devices has sparked lab‐on‐chip technologies with applications in chemistry, physics, biology, and medicine.^[^ ^100^ ^]^ Creating testing kits containing preloaded reagents for varying applications is currently a significant area of focus, holding the potential for life‐saving diagnostics that can be deployed with minimal other equipment. Such devices may allow for the minimization of equipment required for in‐the‐field testing, enabling testing to be carried out at the point of care, before significant delays could occur through the shipment of samples to alternate testing facilities.^[^ ^101^ ^]^ Microfluidic channels are also being used as interfaces for microneedle arrays, as well as self‐enclosed microfluidic channels.^[^ ^102^ ^]^

Other significant devices such as microfilters have been produced using OIHs such as SZ2080. These micrometer pore size microfilters, produced via in situ polymerization inside a closed channel microfluidic device displayed the ability to filter red blood cells from whole diluted blood, with the aim to creating plasma separation filters (Figure 8D). These filters displayed robustness to repeated use and high back pressure cleaning operations, displaying the advantage of high precision, low shrinkage hard resins in TPP applications.^[^ ^103^ ^]^

#### 4.3.4 Organic/Inorganic Hybrid‐Based Optics

Optical applications of OIH's have been recently demonstrated in the fabrication of microresonators (Figure 8E).^[^ ^104^ ^]^ These optical devices, used for investigations into light–matter interactions, have been produced in a myriad of materials and geometries, using the accuracy and optically smooth resolution capabilities of TPP to achieve novel devices. These devices have been reviewed by Otuka et al. where a comprehensive description of both the materials used and photophysics behind microresonators is discussed.^[^ ^41^ ^]^

### 4.4 Epoxides

**Figure 9:**
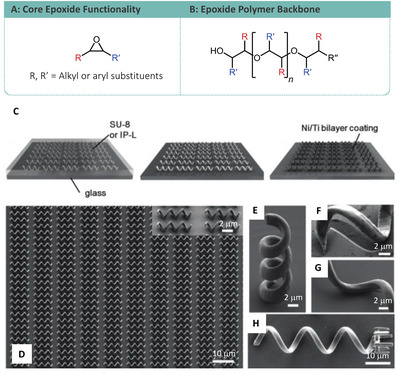
A) Core epoxide monomer functionality. B) Epoxide polymer backbone. Helical microswimmers constructed using SU‐8 epoxide‐based photoresist and IP‐L acrylate‐based resist. C) Construction methodology involving TPP followed nickel/titanium coating. D) An array of helical microswimmers. E) Vertically printed SU‐8 microswimmer. F+G) Close‐up of shadowing on the substrate arising due to the evaporation process associated with the coating process. H) Microswimmer functionalized with a holder for carrying microparticles. (C)–(H) Reproduced with permission.^[^ ^107^ ^]^ Copyright 2022, Wiley.

Epoxy resins are a long‐standing staple of industry where their application as rigid polymers within adhesives and coatings is ubiquitous.^[^ ^105^ ^]^ Epoxides are somewhat less common within TPP due to the requirement for postprocessing baking steps to develop microstructures, with acrylates being more common due to their ease of processing. However, the low degrees of shrinkage upon polymerization associated with epoxides may appeal to the generation of microstructures in which exact dimensions are necessary. Epoxides undergo ring‐opening cationic polymerization (**Figure** **9**A,B) and hence instead of radical initiators, are reliant on photoacid initiators. The nucleophilic attack of epoxide monomers on the cationic carbon typically occurs on the most substituted carbon, with electron‐withdrawing substituents adding to the susceptibility of the breaking of the epoxide ring carbon‐oxygen bond, demonstrated in the increased reactivity of tetracyano epoxide.^[^ ^106^ ^]^ However, despite the processing requirements a commercially available epoxide based photoresist, known as SU‐8, has been used in many applications including medical and microfluidic devices.

#### 4.4.1 Epoxide‐Based Microswimmers

Helical microswimmers have also been constructed using the more rigid epoxide‐based SU‐8 photoresist. The design of the micro‐swimmers, designed as three‐turn helices with a microparticle carrying cage, was possible due to the ability of the TPP process to access arbitrary shapes, which would be technically difficult if not impossible by traditional processing methods. The helical microswimmers were printed followed by coating with a nickel‐titanium bilayer by electron beam evaporation, enabling them to be magnetically driven within the media (Figure 9C). The metallic bilayer had the added benefit of increasing the structures biocompatibility, tested against mouse myoblast (C2C12) cells, exhibiting unaffected proliferation compared to untreated glass coverslips. The microswimmer design was optimized with a 65° helical angle for swimming in aqueous media influenced by a high frequency rotating magnetic field (Figure 9D–G). The microparticle carrying capability of the microswimmers was also demonstrated with the “pick‐up and carry” ability of 6 µm polystyrene microparticles (Figure 9H).^[^ ^107^ ^]^ This may further the use of microswimmers as drug delivery agents, able to carry microparticles loaded with therapeutics to areas previously inaccessible without invasive surgery.

Osmotically driven microfluidic swimmers have also been produced based on the use of bioanode and biocathodes separated by a nonconductive polymer. The main constituent used in these microswimmers was SU‐8, layered with three individual components which upon TPP resulted in a three‐unit device. The biocathode and bioanode elements drive osmotic flow through the hollow center of the device, capable of propelling the device at 100 mm s^−1^ velocities (Figure 2).^[^ ^108^ ^]^

#### 4.4.2 Epoxide‐Based Microfluidic Devices

Microfluidic devices have been produced using SU‐8 photoresist. However, due to regularly induced overexposure at rapid writing speeds, a modified resist consisting of SU‐8 epoxide oligomers purchased without the initiator has been demonstrated in microfluidic designs and this system contains the custom photoacid generator 9,9‐diethyl‐[bis‐(styrytriphenyl sulfonium hexafluoro phosphonate)]fluorene as the initiator. The modified resist allowed for the generation of high aspect ratio channels without overexposure even at high laser doses. The resist was used to produce epoxide master templates, consisting of the positive impressions of the intended channels to be created. The final production of the device was achieved via polydimethylsiloxane (PDMS) casting of the micropatterned positive impression channels and was noted to achieve high fidelity for a range of arbitrary channel shapes.^[^ ^109^ ^]^

## 5 Modification Strategies for TPP Resins

**Figure 10:**
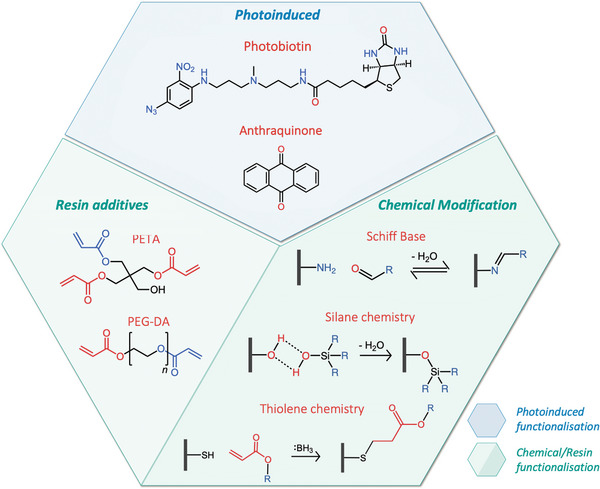
Photoinduced, chemical modification and resin additive methods for the functionalization of polymeric surfaces and materials.

While commercially available TPP resists are highly prevalent in the literature, several examples of covalently modified and custom formulated resist have been designed to tune the physical and chemical properties of microstructures. These modifications were achieved by adding custom formulated monomers to existing resists, postpolymerically modified with specific functional groups, and attached to reactive handles within the surface of a resist (**Figure** **10**).

### 5.1 Acrylates

Anthraquinone has been used in several instances as a photoinitiator for TPP, including a water‐soluble formulation.^[^ ^110^, ^111^ ^]^ As such, the use of modified anthraquinone derivatives for postpolymerization modifications does not require other radical initiators. Anthraquinones can form radicals on exposure to light and abstract alkyl protons from surfaces, forming a pair of radicals. Rearrangement of the carbon anthraquinone radical to form an oxygen radical allows for the combination of radicals and ether linkage of anthraquinones to a surface (Figure 10).^[^ ^112^ ^]^ The use of this method was demonstrated in the production of IP‐L 780 microstructures modified using anthraquinone conjugated to a short peptide chain. The increased hydrophilicity of the resulting microstructures was used to enhance the penetration of the mucus barriers of the lower intestinal tract.^[^ ^113^ ^]^ This method could feasibly be adapted and applied to any alkyl polymer. For attaining custom functionalization, there are well‐reported modification strategies for anthraquinone, allowing access to 2,7‐anthraquinones through the dimethyl derivative using alkyl halogenation, as well as countless derivatives through Diels–Alder reactions of substituted dienes with methyl 4‐oxo‐4‐phenyl‐but‐2‐ynoate followed by sulfuric acid‐mediated oxidation.^[^ ^111^, ^114^ ^]^

### 5.2 Thiol–Ene Chemistry

To our knowledge, there are no commercially available thiol–ene photoresists for use within TPP, however, thiol–ene radical chemistry is routinely applied as a powerful tool for creating robust, high‐resolution chemically modifiable microstructures. Initial use of thiol–ene type resists was demonstrated in the production of woodpile photonic crystals, fabricated using thiol‐containing monomer pentaerythritol tetrakis (mercapto acetate) and vinyl‐containing monomer pentaerythritol tetraallyl ether (PETE).^[^ ^115^ ^]^ The photoresist used the radical photoinitiator 7‐diethylamino‐3‐thenoylcoumarin and mixed using dimethylacetamide. Interestingly, the use of this photoresist involves components that are all commercially available, except for PETE, which was synthesized in a single step. The FTIR spectra of the resulting polymerized structures were compared to the spectra of the unpolymerized photoresist, and a lack of vinyl peak was noted in the polymerized material compared to the monomeric species, with only a reduction of the thiol peak. The residual thiol groups of the surface have been exploited using thiol–Michael addition to attach fluorescent and bromine functionalized vinyl substituents, confirmed using X‐ray photoelectron spectroscopy (XPS) and laser scanning microscopy (LSM).^[^ ^115^ ^]^ Another successful example of modified acrylate photoresists prepared by thiol–ene chemistry was demonstrated via the stoichiometric reaction of monofunctional thiols with PETA to form monofunctionalized triacrylate monomers for use in TPP. Next, an NHS–fluorescein dye was conjugated to the surface in a single‐step postpolymerization, demonstrating the potential for this approach to be applied to a wide range of materials.^[^ ^116^ ^]^

Thiol–ene chemistry is a widely applied coupling technique, boasting a vast substrate scope and is achievable under mild conditions, allowing it to be considered as a “click chemistry” reaction. Since thiol–ene chemistry is a widely used coupling methodology, several important review articles covering the application and scope of the method have been published.^[^ ^117^, ^118^ ^]^ In this review, we focus on the radical and Michael addition of thiols to alkenes. Radical thiol–ene chemistry relies on the abstraction of labile thiol proton by a radical initiator, followed by the radical attack on a vinyl monomer to form a carbon radical. Michael addition thiol–ene reaction relies on the nucleophilic attack on the thiol, often performed using catalytic amounts of a base such as a tertiary amine, producing a thiol anion (Figure 10). The thiol anion can then undergo a nucleophilic attack on an activated vinyl group.^[^ ^118^ ^]^ While the substitution possibilities of vinyl and thiol monomers for thiol–ene type reactions is seemingly endless, reaction rates and kinetics of the reactions are often affected by choice of substituting groups.

By altering the physical and mechanical properties of TPP polymers, flexible materials based on thiol–ene PDMS hybrid materials have been developed for applications in designing waveguides. Here, thiol–ene crosslinking was selected due to the high hydrolytic and thermal stability of the resultant polymers aiding its use where thermal degradation is of concern (such as printing the circuit). The synthesis of the thiol monomer used in this example was achieved using chlorine functionalized disiloxane and thiourea followed by TPP initiated crosslinking with *tris‐*(vinyl‐dimethyl siloxy)‐methylsilane mixed with PDMS.^[^ ^119^ ^]^

### 5.3 Hydrogels

The significant drawbacks of commonly used hydrogels are their mechanical stability and processing capabilities, with many displaying significant deformation from the intended design after TPP. Crosslinking vinyl substituents into a hydrogel structure such as the addition of PETA to PEG‐DA can increase the mechanical stability of their structures with little to no effect on the biocompatibility of the material. From this advancement, the field has shifted beyond the simple addition of crosslinking agents to hydrogel oligomers to modifying hydrogels themselves. For example, gelatin‐B has been modified with methacrylate moieties through the activated ester amidation of the carboxylic acid groups with amine functionalized methacrylates.^[^ ^120^ ^]^ This methodology, using the formation of activated esters from carboxylic acids is a popular condensation technique that uses carbodiimides, uronium, and guanidinium salts.^[^ ^121^ ^]^ This method relies on forming an activated ester through the nucleophilic attack of the deprotonated carboxylate on the coupling reagent, creating an excellent leaving group. Countless other coupling methods between amines and carboxylic acids exist, such as the use of anhydrides and the use of acid chlorides. This method presents ample opportunity for alternative functionalization of the gelatin backbone using varying anhydride or amine compounds.

### 5.4 Organic/Inorganic Hybrids

OIHs have been modified for potential application in the production of 3D patterned biological sensors with biomolecules such as biotin for sensing applications when sample volumes must be kept to a minimum. One example involves printing OIH surfaces with ORMOCER, a commonly used material in TPP methodologies for production of biological interface devices and robust microstructures such as microneedles. The resultant OIH surfaces were capable of being modified by photobiotin photolysis using a KrF excimer laser.^[^ ^122^ ^]^ Here, the photobiotin was applied to the TPP polymerized ORMOCER surface in dilute aqueous ethanol followed by vacuum drying and photolysis, yielding surface‐bound biotin. The successful functionalization of the surface was measured through fluorescence microscopy after incubation with fluorescent streptavidin, which selectively binds to the biotin coating on the OIH surface.^[^ ^122^ ^]^ The use of streptavidin binding to biotin is a commonly used coupling technique, with use cases for both fluorescent labeling as described above down to DNA purification methodologies.^[^ ^123^ ^]^

### 5.5 Epoxides

SU‐8 is a common epoxide‐based photoresist for TPP and while few examples are available for their SU‐8 microstructure modification, modification strategies have been applied to SU‐8 polymeric materials produced using UV photomask lithography. Typical acid catalyzed epoxide ring‐opening reactions liberate a hydroxyl group with the possibility of adding a nucleophile to the carbocation formed in the process. This opens the opportunity for using the hydroxyl group formed in polymeric materials for further modification strategies such as silane chemistry or esterification with activated esters (Figure 10). This also offers the ability for a nucleophile to add at the carbocation, allowing for countless possible changes to be made to the surface chemistry, ranging from the addition of alkoxy groups from alcohols to further addition of hydroxyl groups using water as a nucleophile.

Hydroxyl groups have been used previously to modify polymeric surfaces using silane chemistry (Figure 10), such as the introduction of amine groups to the surface using a silane coupling reagent 3‐aminopropyltriethoxy silane (APTES) diluted in isopropyl alcohol.^[^ ^124^ ^]^ Modification of TPP produced microstructures by similar means followed. The group noted the various methods for producing surface‐bound hydroxyl groups from residual epoxide rings, noting that sulfuric acid treatment for prolonged periods degraded and deformed fine features of microstructures and instead opted for cerium(IV) ammonium nitrate (CAN) catalyzed with nitric acid. On the treatment of SU‐8 structures, increased incubation time with CAN resulted in a greater surface density of hydroxyl groups. Further examples then employed APTES to generate surface‐bound amino groups, which were further exploited through activated ester binding of sulfo–NHS–biotin for the selective binding of streptavidin and the ionic binding of citrate coated gold nanoparticles to the surface‐bound amines.^[^ ^125^ ^]^ This work displays the utility of SU‐8 polymers as modifiable scaffolds through functionalized silanes, activated esters, or through novel ionic interactions with charged surface‐bound amines.

## 6 Summary and Outlook

TPP has become widely adopted in cell biology, optical engineering, and medical device development. TPP boasts an unparalleled ability to produce micro‐ and nanostructures with resolutions and arbitrary geometry beyond the capabilities of other subtractive and additive manufacturing approaches. Extensive work has been undertaken using commercially available photoresists, using rigid materials such as acrylates, epoxides, and OIHs to produce optically smooth microstructures for applications in micro‐optics and biocompatible materials for the production of next‐generation medical devices. In addition, significant attention has focused on applying soft hydrogel materials in cell and chemical biology for advancing cell growth, imaging, and tissue engineering technologies. The advancement of these technologies has led to new possibilities in cellular imaging by mimicking natural cell environments by inducing 3D or structured cell growth and the advanced tissue architectures possible when coupled with techniques such as single‐cell LIFT. While commercially available 2PP polymeric materials have been extensively researched and combined with, for example, hydrogel/ORMOCER structures for cell scaffolding, as of yet, little overlap exists between synthetic chemistry, materials science, and polymer chemistry. An exception here is the application of thiol–ene chemistry, being one of the few prominent examples to engage all three areas. However, these methods are constantly evolving to further tailor the structures produced, moving down from the micro‐ and nanoscales down to the molecular, with significant advances in TPP materials production in the past decade. Structures can now be produced with increased chemical functionality, with fluorescent woodpile photonic crystals and waveguides using thiol–ene chemistry, as well as carbodiimide coupling and silane chemistry finding applications in conjunction with existing monomers. With these current advances in surface chemistry, coupling and analysis methods, the production of molecularly tailored surfaces for state‐of‐the‐art applications is now coming to the fore in existing materials technology.

While this TPP has gained much exposure in the fields of biology, chemical‐biology, and novel device development, synthetic chemistry is only beginning to be introduced as a functional tool for the tailoring of polymeric microstructures in pre‐ and postfunctionalization strategies. Thus, significant opportunities exist for expanding modification strategies of polymeric materials with extensive possibilities to produce devices with chemical functionality through facile conjugation reactions and “click” chemistry. Furthermore, little work exists to produce localized functionality on a single device, where the high precision capabilities boasted by TPP have not been taken advantage of. However, simple dual material devices such as PEG‐DA/Ormocomp cell scaffolds do offer successful examples to build upon. Overall, there is now extensive scope to produce custom formulated resins using systematic and multistep approaches for the creation of next‐generation multifunctional microscale polymeric devices.

Currently, the primary limitations of TPP arise due to its processing scalability, long print times, and the instrument expense. Scalability and high‐throughput production are two of the main limiting factors for TPP, with only microscale production possible on most TPP systems. However, to adapt TPP to the industrial scale, this throughput issue has been the focus of extensive research, with successful examples using digital micromirror devices (DMDs), multifoci scanning, microchip lasers, and resonant mirror scanners producing large field printing capabilities for TPP.^[^ ^126^, ^127^, ^128^, ^129^ ^]^ While not yet widely adopted, commercial systems offering similar capabilities are now entering the market.

## Conflict of Interest

The authors declare no conflict of interest.
